# Novel application of metagenomics for the strain-level detection of bacterial contaminants within non-sterile industrial products – a retrospective, real-time analysis

**DOI:** 10.1099/mgen.0.000884

**Published:** 2022-11-24

**Authors:** Edward Cunningham-Oakes, Tom Pointon, Barry Murphy, Stuart Campbell-Lee, Thomas R. Connor, Eshwar Mahenthiralingam

**Affiliations:** ^1^​ Microbiomes, Microbes and Informatics Group, Organisms and Environment Division, School of Biosciences, Cardiff University, CF10 3AX, UK; ^2^​ Department of Infection Biology and Microbiomes, Institute of Infection, Veterinary and Ecological Sciences, University of Liverpool, Liverpool, L69 7ZB, UK; ^3^​ Unilever Research and Development, Port Sunlight, Bebbington, CH63 3JW, UK; ^4^​ Arxada, Crumpsall Vale, Blackley, Manchester, M9 8GQ, UK

**Keywords:** microbial contamination, metagenomics, strain identification, bioinformatics, metagenome binning

## Abstract

The home and personal care (HPC) industry generally relies on initial cultivation and subsequent biochemical testing for the identification of microorganisms in contaminated products. This process is slow (several days for growth), labour intensive, and misses organisms which fail to revive from the harsh environment of preserved consumer products. Since manufacturing within the HPC industry is high-throughput, the process of identification of microbial contamination could benefit from the multiple cultivation-independent methodologies that have developed for the detection and analysis of microbes. We describe a novel workflow starting with automated DNA extraction directly from a HPC product, and subsequently applying metagenomic methodologies for species and strain-level identification of bacteria. The workflow was validated by application to a historic microbial contamination of a general-purpose cleaner (GPC). A single strain of *Pseudomonas oleovorans* was detected metagenomically within the product. The metagenome mirrored that of a contaminant isolated in parallel by a traditional cultivation-based approach. Using a dilution series of the incident sample, we also provide evidence to show that the workflow enables detection of contaminant organisms down to 100 CFU/ml of product. To our knowledge, this is the first validated example of metagenomics analysis providing confirmatory evidence of a traditionally isolated contaminant organism, in a HPC product.

## Data Summary

The authors confirm that all supporting data, code and protocols have been provided within the article. Illumina raw sequence reads have been deposited in the European Nucleotide Archive (ENA) under ENA project accession number PRJEB53377.

**Impact Statement**

Home and personal care (HPC) product microbiology is an area where the rapid and early detection of contamination is essential to protect the health of the consumer, and minimise economic and reputational damage to the industry, if contaminants are pathogenic microbes. In contrast to the food industry, where sequencing is routinely used to monitor outbreaks of organisms such as *Salmonella *, *Escherichia coli* (STEC), and *Listeria monocytogenes *, the use of genomics for bacterial surveillance in the HPC industry is relatively unexplored. The metagenomics workflow presented in this study benefits both industry and the consumer, by providing risk understanding, and comparative data for long-term surveillance of industrial contamination. Moreover, the study also demonstrates the ability to expand the traditional microbiology toolkit currently used by the HPC industry, in a way that has the potential to improve speed, taxonomic accuracy and sensitivity of detection.

## Introduction

Industries that manufacture home and personal care (HPC) products, such as body wash and dish wash liquid, are major users of antimicrobial compounds in the form of preservatives [1]. Preservatives are used because HPC products are largely based on water and other components such as nutrients, oils and proteins that readily support the growth of microorganisms [2]. Regulatory guidelines state that these products are not expected to be sterile, but should neither contain specified microorganisms with the potential to affect product quality and consumer safety, nor permit the growth of any non-specified microorganisms [3]. Preservatives are therefore used to limit microbial growth in HPC products and preserve the shelf life of the product [3] which could potentially be contaminated at multiple stages, including inadvertently during manufacture, and during consumer use via repeated microbial challenge from the home environment and skin flora [4, 5].

However, the HPC industry is facing increasing pressure in the face of regulatory, safety and public relation pressures, which limit the available palette of preservative chemistries [3, 6]. Intrinsically preservative-tolerant Gram-negative bacteria, which may occasionally overcome product preservation and cause contamination during manufacture or long-term storage, add further complexity to the preservation challenge [7]. Bacterial species of the genera *Pseudomonas* and *Burkholderia* have also been shown to acquire a tolerant phenotype following prolonged exposure to preservatives [1, 8]. ISO 11930 and European Pharmacopoeia challenge testing methods require an absence of *Pseudomonas aeruginosa* from non-food products, while *Burkholderia* are known opportunistic pathogens in cystic fibrosis patients and immunocompromised individuals [9, 10]. Thus, early detection of bacterial contamination is critical to protect consumers and industry.

Traditionally, the identification of organisms has revolved around cultivation-dependent methods that assess phenotype [11]. This involves subjecting microorganisms to a plethora of biochemical and physiological assays, observation of growth characteristics on selective and differential media, and assessing antibiotic susceptibility and microscopic morphology [12, 13]. These and other culture-based techniques are currently the mainstay of identification in industrial settings, in part due to their ease of use, but also owing to acceptance and regulatory requirements [3]. An increasing number of non-colony-forming unit (CFU) detection methods such as ATP-bioluminescence, impedance, flow cytometry and PCR-based assays have been introduced to areas such as food microbiology in order to screen for contamination [14–16]. While these methods can be complementary to CFU-based alternatives [17], techniques such as ATP-bioluminescence still require a culturing step to reliably distinguish between different microorganisms [18]. These methods also do not resolve bacterial identification to the strain-level which, in the context of the changing landscape of HPC manufacturing, is useful for surveillance of contamination.

The use of cultivation-independent techniques is relatively unexplored territory in the HPC industry. However, the aforementioned research, alongside a desire for detection limits of 100 CFU/ml or less [3], suggest that a cultivation-independent detection methodology that can be implemented in a high-throughput manner would be desirable in an industrial environment. Metagenomic approaches also have the benefit of generating genomic DNA sequence data which can provide accurate taxonomic identification of microbial contaminants, a gap which needs to be addressed in the international product recall databases which report spoilage incidents [7]. Genomic-level taxonomy is also vital for the accurate resolution of *Burkholderia cepacia* complex contaminants which belong to novel species groups such as *Burkholderia aenigmatica *, for which techniques such as 16S ribosomal RNA (rRNA) gene sequencing provide insufficient resolution for accurate identification [19]. With extensive DNA databases in place, genomic identification also enables strain-level identification of bacterial contaminants [19].

Herein, we describe a novel cultivation-independent workflow, using a one-step automated DNA extraction method to extract directly from a HPC product, followed by next-generation sequencing for species and strain-level identification of bacteria. The workflow was applied to a historic microbial contamination of a general-purpose cleaner (GPC). In parallel, traditional, cultivation-based microbiology followed by genome sequencing of the isolated bacterium was used to validate our metagenomic data and show that the workflow offers an improvement over existing methodology. Finally, we also provide evidence that the metagenomics protocol enabled detection of the contaminating bacterial strain down to 100 CFU/ml. This unique and direct application of metagenomics for detection of HPC product contaminating microorganisms has multiple benefits for the surveillance and understanding of preservative-tolerant bacteria which occur as industrial contaminants.

## Methods

### Cultivation-dependent and independent DNA extraction

**Fig. 1.**
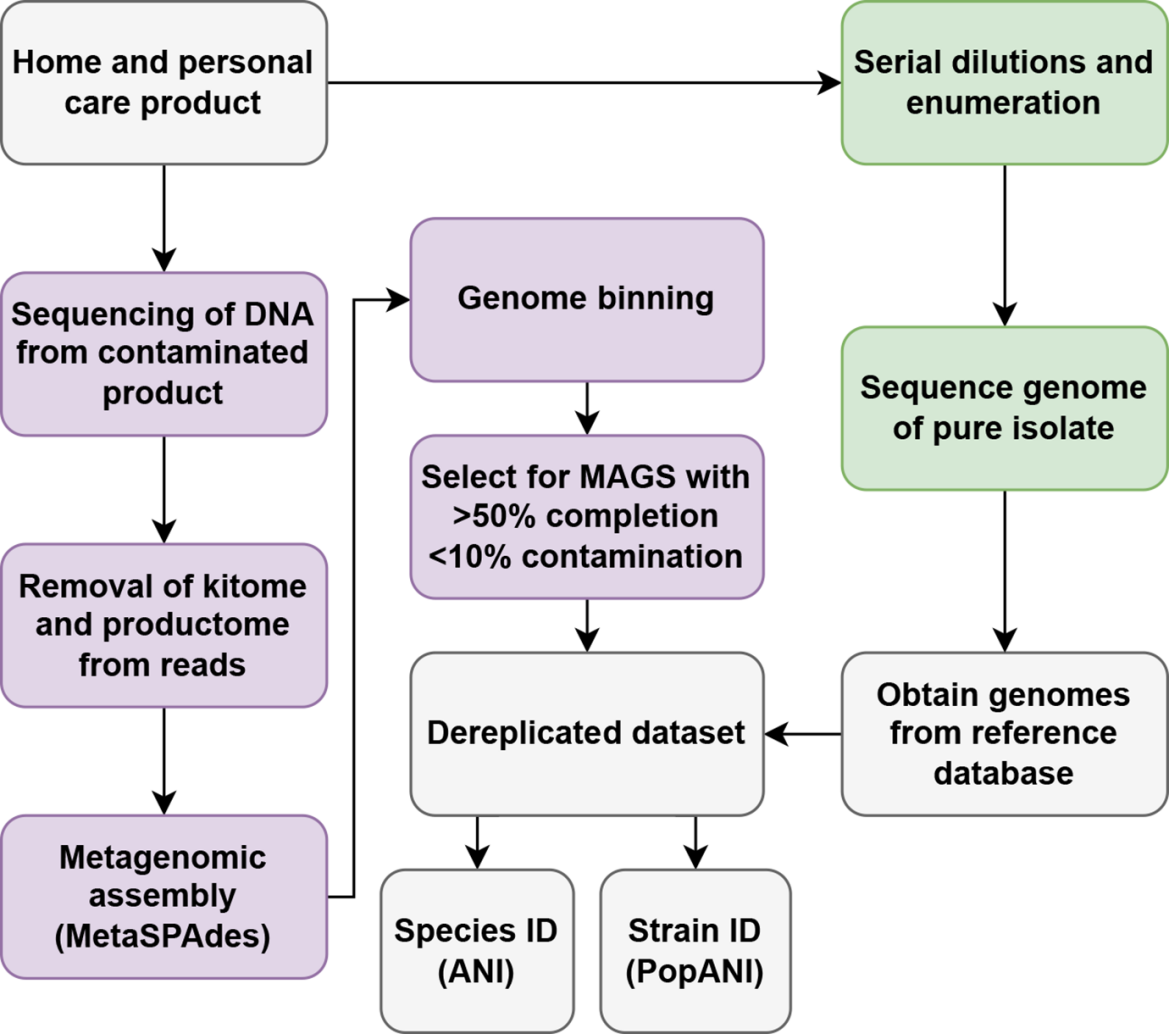
Workflow used for cultivation-dependent and cultivation-independent detection of contaminant organisms in this study. Genomes chosen from reference databases can be changed based on known organisms of concern, *a priori* knowledge, or site specific needs. Parts of this workflow that are exclusive to cultivation-independent (purple) and traditional microbiology (green) arms of analysis are highlighted.

A workflow enabling parallel metagenomic sequencing and bacterial cultivation analysis of a HPC contaminant was developed (Fig. 1). Bacterial isolates were cultivated by plating serial dilutions in sterile deionised water (to 10^−7^; 50 µl drops) of the GPC onto a nutrient-rich agar, tryptic soya agar (TSA), and a minimal agar, Reasoner’s 2A (R2A) agar, to account for the presence of slow growing organisms. The same dilution series enabled colony counts to be performed and enumeration of the detection limits of the metagenomics in comparison to cultivation (see below). Colonies isolated from TSA were inoculated into 3 ml tryptic soya broth for overnight culture (18 h), while colonies from R2A were inoculated into 3 ml R2A broth. Overnight cultures were pelleted, then suspended in 300 µl of the lysis agent Guanidinium isothiocyanate (4M concentration), before extraction. The GPC dilution series used for counts was also mixed in a 1 : 1 ratio with Guanidinium isothiocyanate, before 400 µl was transferred for cultivation-independent DNA extraction. A series of dilutions were also performed using a non-contaminated GPC, and DNA extraction cartridges from the same kit used to extract both cultures, and contaminated GPC. The dilutions and extractions were performed identically to those performed for the contaminated product and served as controls to enable the removal of background DNA within the generic product formulation or extraction kit (referred to herein as the ‘productome’ and ‘kitome’ respectively).

In order to validate the assumption that *Pseudomonas *-assigned reads from the contaminated product were distinguishable from those in the kitome and productome, raw reads were annotated using Kraken2 (v2.1.2) [20]. Assignments were made using a custom database, containing RefSeq complete genomes and proteins for archaea, bacteria, human, fungi, viruses, plants, and protozoa, as well as RefSeq complete plasmid nucleotide and protein sequences, and a subset of the NCBI UniVec database. A confidence threshold of 0.1 was set for read assignments, and reports were generated for downstream biom file generation. Kraken-biom (v1.0.1) [21] was used to generate a biom file from Kraken2 report files. Biom (v2.1.6) [22] was then used to assign tabulated metadata to this biom file. Ordination plots representing taxa assigned to reads were then visualised using Phyloseq (v1.36.0) [23], with non-metric multidimensional scaling (NMDS) and Bray-Curtis dissimilarity.

Sample processing was performed during: (i) the week of contamination (week 1), and (ii) repeated 2 weeks post-contamination (week 3); this was used to detect whether the contamination signatures were stable or increased over time. All culture and product DNA extractions were then sequenced on a NovaSeq 6000, using 150 bp paired-end sequencing by a commercial sequencing provider (Novogene, Cambridge, UK) as described previously [19].

### Establishing a reference database of complete *Pseudomonas* genomes

Initial 16S rRNA gene sequence-based identification of the bacterial contamination of the GPC highlighted *P. aeruginosa* as a potential cause; this analysis was performed by neutralising the product in peptone-tween, before plating onto TSA. Once growth was recovered, colonies were then washed in nuclease-free water, and then suspended in absolute ethanol. This suspension was then sent to a third party for DNA extraction and 16S rRNA identification. Based on this, we opted to generate a reference *Pseudomonas* genome database, against which we could compare both the genomes from culture, and metagenome-assembled genomes (MAGs). This database was generated by obtaining all complete genomes available from The Pseudomonas Genome database [24] (*n*=612). This reference database was further simplified by using dRep (v3.0.0) [25] to identify redundant genomes within the dataset, leaving only representative genomes (*n*=288). Finally, sequencing reads generated from isolated pure bacterial colonies on week 1 and week 3 were used to generate draft assemblies of the contaminant organism, using SPAdes (v3.15.3) [26] in isolate mode. The draft assemblies were then corrected using Pilon (v1.24) [27] and dereplicated using dRep, resulting in one representative genome, from colonies grown on TSA at week 3. This representative draft genome was amalgamated with the simplified *Pseudomonas* Genome database genomes to make the final reference database (*n*=289). To confirm that dereplication to a single representative genome was correct, Average Nucleotide Identity (ANI) analysis was also performed using PyANI (v0.2.11) [28] with MUMmer as an alignment method (ANIm). This enabled confirmation that the draft genomes generated from culture at each timepoint were, at a minimum, identical species (95 % or greater nucleotide identity in comparison to each other).

### Detection limits of metagenome binning for species-level contaminant identification

In order to standardise analysis as far as possible, we made use of the MetaWRAP (v1.3.2) pipeline [29], which functions as a wrapper around multiple utilities for metagenome analysis. The pipeline was deployed in a dedicated conda environment, using the ‘manual installation’ guide (see https://github.com/bxlab/metaWRAP). For each control dilution, decoy metagenomes were assembled using MetaSPAdes (v3.15.3) [30]. These assemblies were then used with the MetaWRAP ‘read_qc’ module to remove any productome or kitome-aligned reads from the contaminated GPC reads (e.g. the 10^−1^ control dilution was used to remove kitome reads from the 10^−1^ dilution of the contaminated GPC). The remaining reads from the contaminated GPC were then assembled using MetaSPAdes, before being binned with the ‘binning’ module of MetaWRAP, using CONCOCT [31], MaxBin 2.0 [32] and MetaBAT 2 [33] as binning methods. The quality of any resulting bins was then assessed using CheckM (v1.0.12) [34]. Any bins that fell below the threshold required for a medium-quality draft MAG [35] (over 50 % completion and less than 10 % contamination), were removed, using the ‘bin_refinement’ module of MetaWRAP. All bins were then compared to the simplified *Pseudomonas* genome database using FastANI (v1.32) [36] to run a faster, less-computationally intensive analysis to identify the most related genomes to the contaminant organism. This analysis was then used as a basis to obtain the genome for appropriate type strains. Final species identity was then confirmed by comparing all binned metagenomes and culture genomes to the identified type strains, using PyANI.

### PopANI detection limits for cultivation-independent strain-level identification

We aimed to determine whether the cultivation-independent workflow devised was sufficient to i) accurately strain-type a contaminant organism via recruitment of metagenomic reads to the correct reference genome and ii) determine the detection limits of this methodology. To achieve this, the productome and kitome-subtracted reads for all dilutions were then recruited to our *Pseudomonas* genome database (described below). Reads were first mapped to all reference genomes using Bowtie 2 (v2.4.5) [37]. The genomes of identical strains were then identified within the database by using inStrain (v1.3.4) [38] and population ANI (popANI) metric, which uses a stringent cut-off of 99.999 % as the definition of an identical strain. Several metrics were generated by inStrain as follows: Coverage overlap= percentage of bases that are either covered or not covered in both of the profiles (Formula=length[coveredInBoth] / length[coveredInEither]); Compared base count=The number of considered bases; popANI=The Average Nucleotide Identity among compared bases between the two scaffolds (Formula = [compared_bases_count - population_SNPs] / compared_bases_count); Compared=percentage of the genome compared. The breadth and depth of coverage in higher-quality samples was assessed by i) using BWA (v0.7.17) [39] with samtools (v1.13) [40] to map to our representative genome from culture (described above), and ii) using the coverage command within samtools to provide coverage statistics.

## Results

### A single bacterial colony type was present in the general-purpose cleaner at >10^4^ CFU/ml

**Fig. 2.**
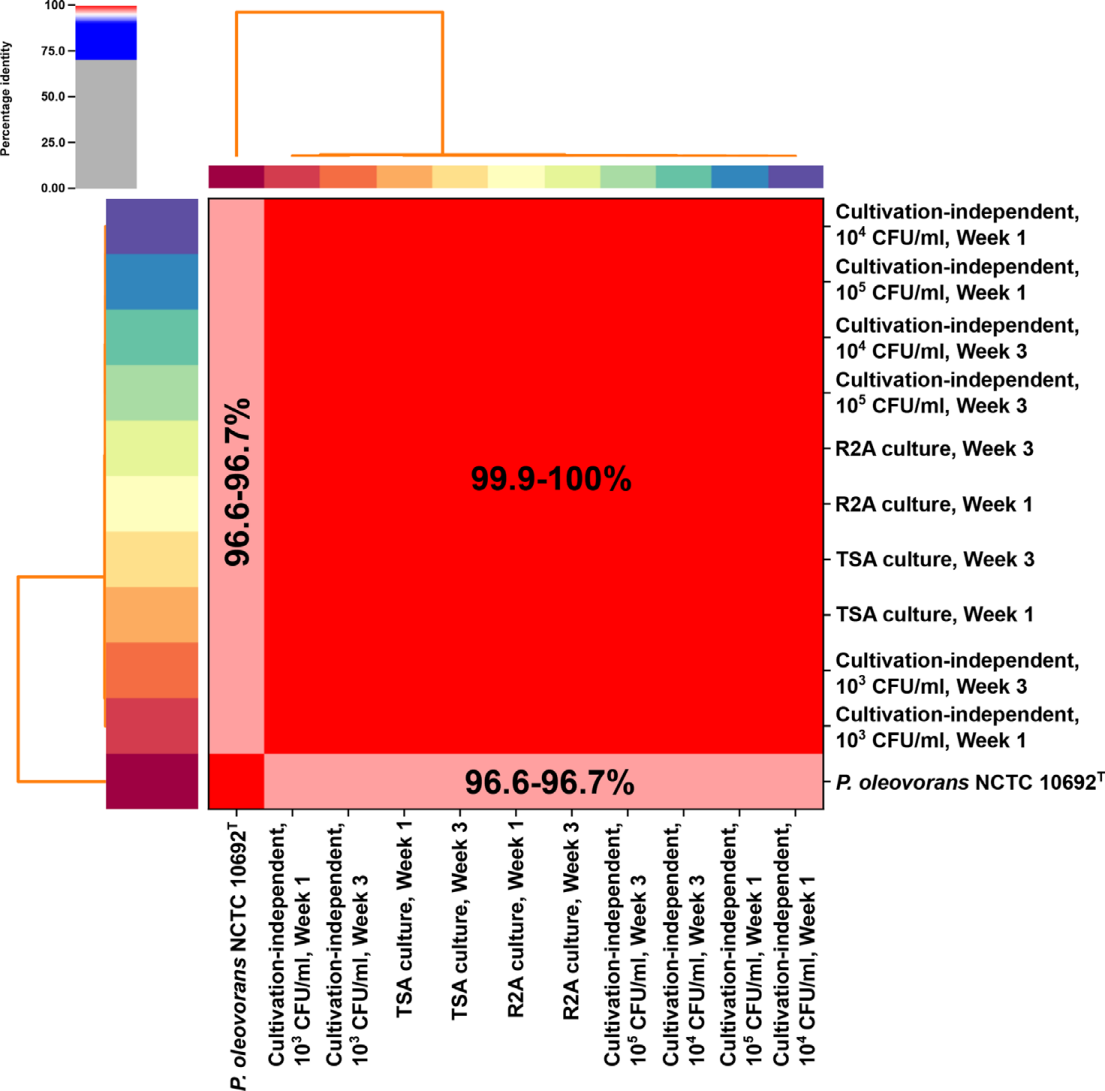
Genomic taxonomy of the HPC contaminant strain. ANIm analysis, showing pairwise comparisons between MAGs, assemblies from culture, and the *P. oleovorans* type strain, NCTC 10692. This analysis shows that i) cultivation-independent MAGs and those assembled from culture-based sequencing data are nearly identical and ii) that the species identity of the contaminant organism was *P. oleovorans *. The darker the shade of red between pairwise comparisons, the higher the percentage shared nucleotide identity. Colours around the outside of the heatmap are used to delineate the positions of individual samples on the heatmap.

The workflow employed both cultivation-independent and growth-based microbiology in parallel (Fig. 1). Traditional microbiology enabled the background level of bacterial contamination within the GPC to be enumerated and morphologically characterised as follows. Counts performed prior to DNA extraction indicated that a single colony type observed on TSA was present in the contaminated GPC at 3.60×10^5^ CFU/ml in week 1, and 2.44×10^5^ CFU/ml in week 3. The second pure morphology observed on R2A was present at 6.52×10^4^ CFU/ml at week 1, and 8.86×10^4^ CFU/ml at week 3. Conversely, nothing was cultivated from the control GPC. Assembly of the genomic DNA reads from the pure cultures on TSA and R2A across the timepoints resulted in four draft genomes of total length ranging from 4.98 to 5.01 Mbp. ANIm confirmed that all the genomes were near taxonomically identical (99.9–100 % identity when compared to each other; Fig. 2). As the organisms isolated on each media type were identical, TSA was taken as the optimal nutrient-rich growth medium for the contaminant organism. As such, all cultivation-independent detection limit calculations in subsequent analyses were made in reference to counts observed on TSA for each time point.

### Unsupervised metagenome binning enabled reliable identification of the contaminant species group down to a presence of 10^3^ CFU/ml

In parallel to the traditional microbial cultivation and enumeration of the bacterial contaminant within the GPC, the novel metagenomic workflow (Fig. 1) demonstrated the following characteristics for the bacterial DNA within the sample. Initial metagenome binning produced bins for dilutions down to 10^1^ CFU/ml across all binning methods. For dilutions beyond this, no bins were produced. Subsequent refinement of bins based on MIMAG standards [34] removed any bins from dilutions containing counts under 10^3^ CFU/ml (see Table 1). The closest matching species for both the pure culture assemblies and MIMAG-quality metagenome bins was a *Pseudomonas oleovorans* genome (Accession: GCF_000953455.1, 96.8–97.0 % identity by FastANI). A subsequent comparison to the type strain genome *P. oleovorans* NCTC 10692 (Accession: GCF_900455615.1) via ANIm confirmed this species identity, which was consistent across all isolated culture genomes and MAGs (96.6–96.7 % identity, see Fig. 2). This also demonstrated that the initial 16S rRNA gene based molecular identification as *Pseudomonas aeruginosa* did not have sufficient resolution for accurate identification of the contaminant.

**Table 1.** Genome completion metrics for MAGs from cultivation-independent DNA samples taken from the contaminated GPC. Key: Timepoint=timepoint at which DNA extraction was performed; Completeness=estimate of MAG completeness, based on presence/absence of single-copy genes; Contamination=estimate of MAG contamination, based on whether a single copy gene appears more than once; Mean coverage breadth=percentage of bases sequenced across the target genome; Mean coverage depth=average number of times the bases of the target genome have sequenced in a sample; GC=MAG GC content, Lineage=estimation of taxonomy, based on lineage-specific marker genes; N50=shortest contig for which longer and equal length contigs cover at least 50 % of the assembly; Size=total MAG size

Dilution	Timepoint	Completeness (%)	Contamination (%)	Mean coverage breadth (%)	Mean coverage depth (fold)	GC (%)	Lineage	N50 (bp)	Size (bp)
NEAT	Week 1	97.51	1.825	98.45	336	62.6	Pseudomonadales	54 936	4 434 208
NEAT	Week 3	96.91	1.501	98.47	355	62.6	Pseudomonadales	53 432	4 359 468
10^1^	Week 1	96.71	1.906	98.31	202	62.6	Pseudomonadales	51 769	4 351 642
10^1^	Week 3	96.5	2.718	97.50	23.8	61.9	Pseudomonadales	19 205	4 801 974
10^2^	Week 1	78.79	0	97.46	14.4	62.7	Bacteria	15 061	4 189 333
10^2^	Week 3	71.89	0	97.03	15.2	62.8	Bacteria	15 503	4 116 775

### PopANI improves cultivation-independent detection limits to 10^2^ CFU/ml

**Fig. 3.**
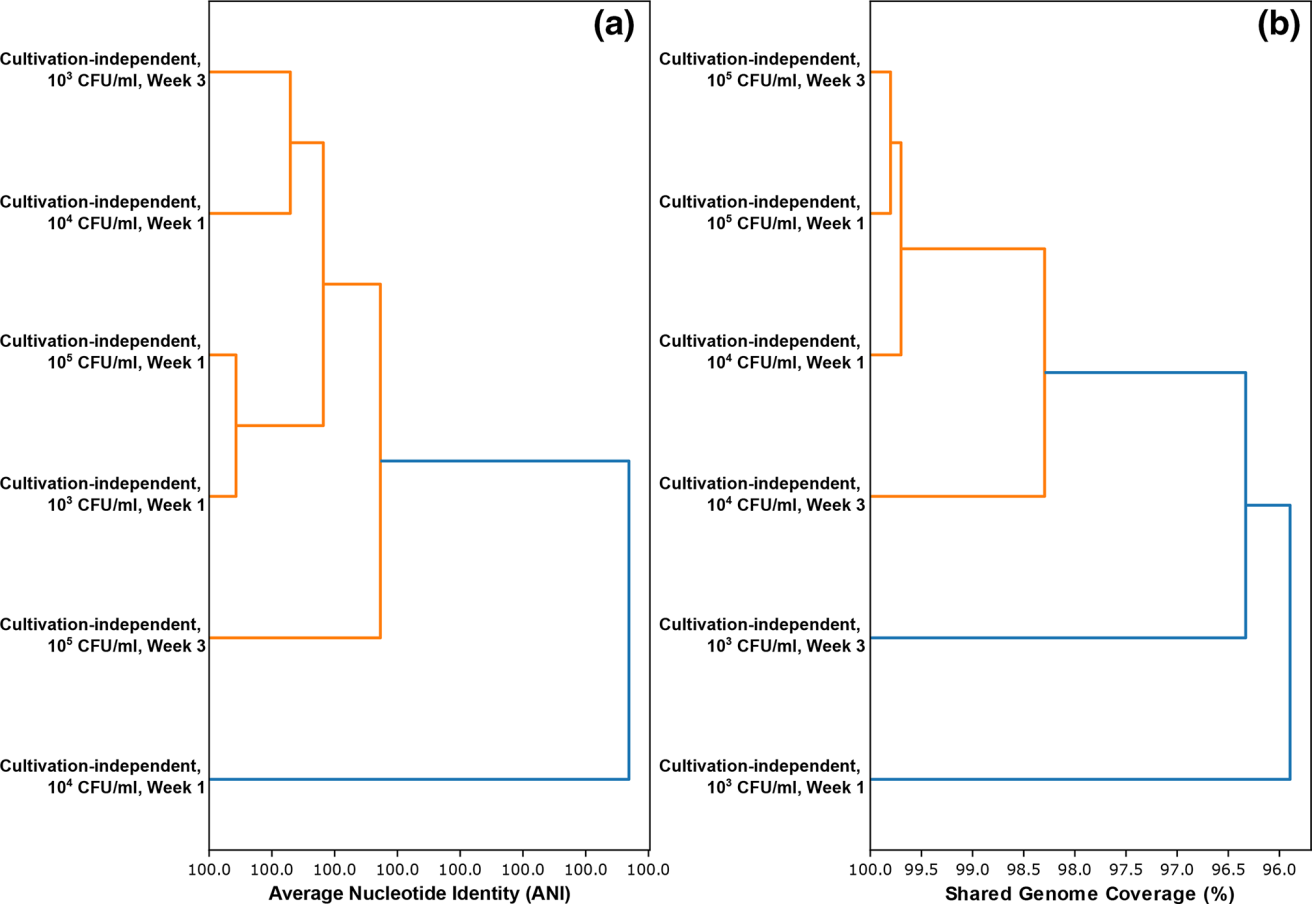
Dendrogram comparing all cultivation-independent samples based on popANI (**a**) and shared bases (**b**). All samples are labelled with both their cell counts (CFU/ml) relative to counts performed on TSA, and time point at which the sample was taken.

Microdiversity analysis using inStrain [37] confirmed that the same isolate cultured from the contaminated GPC could be detected in our cultivation-independent sequencing data (>99.999 % popANI). Data from samples containing ≥10^3^ CFU/ml produced data with a high breadth and depth of coverage (see Table S1, available in the online version of this article). This was also evidenced in the unsupervised binning approach, where coverage was sufficient to produce MAGs from these samples. The organism detected in these samples were nearly identical in terms of popANI and shared a high breadth of coverage between samples (95.05–99.80 % – see Fig. 3 and Table S1). These samples were also placed into a single strain cluster, showing that they were identical to the cultured pure isolate.

The closest matching reference genome using popANI was a *Pseudomonas oleovorans* genome (Accession: GCF_000953455.1), which was also identified as the closest match using binning and ANIm approaches. The popANI approach also enabled the detection of the cultivated isolate at week 1 in the sample containing ~10^2^ CFU/ml (see Table S2). The coverage depth of this sample was comparatively low (2.68×), thus preventing meaningful comparison to samples with higher cell counts and coverage. However, this sample possessed 99.999 % identity to the cultured isolate genome, with a high breadth of coverage (79.20%). Moreover, no matches were seen when compared to other genomes within the simplified *Pseudomonas* database, including the closest matching *P. oleovorans* genome seen for other samples. This signal was absent at the same dilution by week 3. This finding was supported by our ordination analysis, (see Fig. S1), which showed that *Pseudomonas *-assigned reads from the contaminant organism and kitome/productome were distinguishable down to a dilution factor of 10^−3^.

## Discussion

The workflow presented shows that cultivation-independent, automated DNA extraction can be applied directly to HPC products and provides a promising alternative to cultivation-based methodologies for contamination surveillance within preserved industrial products. It can also overcome issues seen in growth-based approaches, which can take up to 7 days for microorganism recovery [41] and are potentially inaccurate depending on the methodology used for identification [19]. Moreover, the use of genomic methodologies such as ANIm and popANI reliably enables detection of down to 10^3^ CFU/ml and identification with species or consensus MAG-level accuracy, respectively.

It is important to note that this analysis does not come without limitations. Firstly, the use of 16S rRNA sequencing from a cultured isolate in this study allowed us to limit the scope of our genomic database to *Pseudomonas *. For this analysis to be truly cultivation-independent, another methodology, such as further interrogation of the k-mer based taxa-assignment results (see Fig. S1), would need to be used to focus analysis towards the likely taxonomy of the contaminant organism. Second, assuming a closely related genome is present within the reference database used, it is possible to extend this detection limit down to 10^2^ CFU/ml, thereby meeting lower detection thresholds required for certain HPC product types [3]. However, this detection limit was only achieved due to the acquisition of the genome from the cultured contaminant and its presence in our database; coverage was too low to identify the closest matching reference species without this. Thus, in instances of low coverage, an exactly matching genome is required to achieve any form of detection. Future work should look to improve coverage from samples with lower cell counts, by investigating methods such as organism-specific amplification steps before sequencing [42]. This would, in turn, enable species identification in the absence of a reference genome that perfectly matches a contaminant organism.

The use of low-input library preparation for both short [43] and long-read [44] sequencing technologies has previously seen success when applied to metagenomics. Such approaches could improve coverage in HPC products with low-level contaminant bioburden, thereby improving the sensitivity of detection [45]. All future work should also seek to validate this workflow further, via application to larger numbers of samples containing a range of organisms of interest to the industry [7], and sample types reflecting the wide variety of products sold to consumers [46]. Methods which attempt to distinguish between viable and non-viable recoveries (e.g. viability PCR) [47], should also be considered. The current workflow does not make this distinction, which would be necessary in cases where background DNA of a related microorganism is abundant. An ideal workflow should also consider emerging cost-saving methodologies [48] to make routine implementation feasible. If the cost could be reduced, then regular surveillance of production facilities could be considered by manufacturers. The workflow could then be used to determine whether there is a build-up of bacterial DNA within industrial product types and could then act as an early warning of a potential manufacturing incident. Implementation will also require this workflow and downstream analysis to be entirely automated, to address both the need for standardisation, and gaps in expertise.

Overall, the HPC industry is undergoing considerable change to reduce environmental impact. This includes working with consumers to incorporate desired natural and milder preservatives and moving away from single-use plastic packaging towards consumer refillable approaches. These changes mean that the detection of microbial contamination will become more vital than ever within the industry, especially since preservative-tolerant bacteria such as *Pseudomonas *, *Burkholderia* and the Enterobacteriaceae are frequently found as problematic HPC product contaminants [7]. The sensitive and accurate metagenomic workflow provided here could be highly beneficial in enabling industry-level surveillance of microbial contamination going forward.

## Supplementary Data

Supplementary material 1

Supplementary material 2
