# Supplementary material for: Novel application of metagenomics for the strain-level detection of bacterial contaminants within non-sterile industrial products – a retrospective, real-time analysis
Source: Microb Genom. 2022 Nov 24;8(11):mgen000884. doi: 10.1099/mgen.0.000884 (PMC9836090; doi:10.1099/mgen.0.000884)
Supplement: Supplementary material 1 [file mgen-8-884-s001.pdf]

Novel application of metagenomics for the strain level detection of bacterial contaminants within non-sterile industrial products – a retrospective, real-time analysis: Supplementary Figure 1

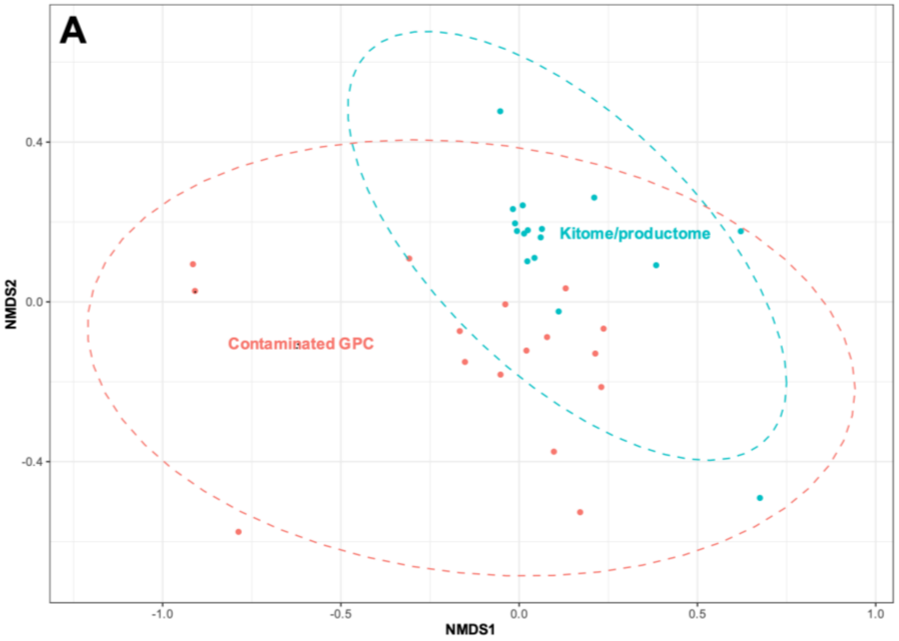

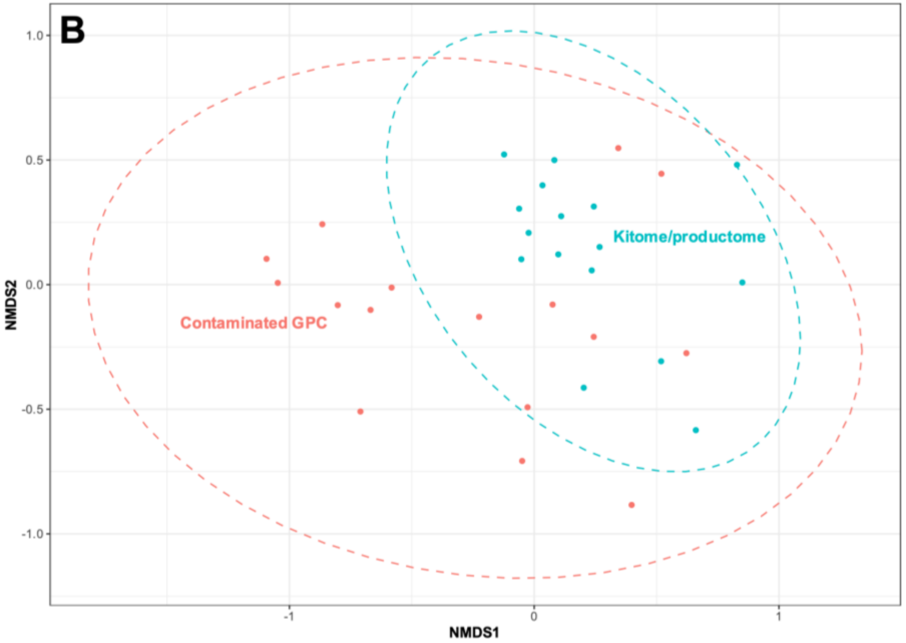

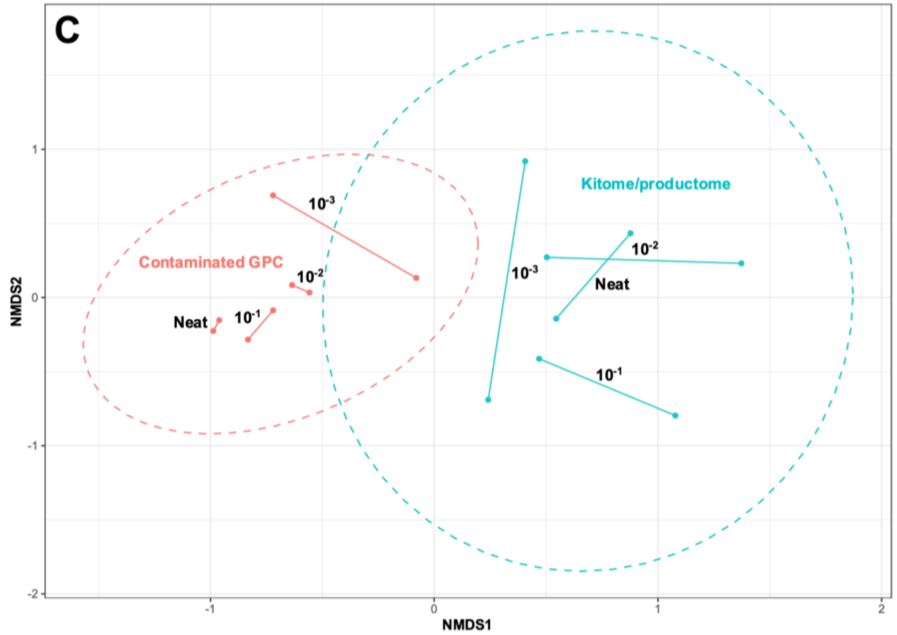

Supplementary Figure 1: NMDS ordination of taxa assigned to metagenomic reads from the contaminated GPC, and kitome/productome (control) samples, on the basis of k-mers. Each point represents all of the k-mers from reads in an individual sample. Ellipses represent the normalised distribution of samples. A: Ordination of reads assigned to all taxa B: Ordination of reads assigned to the genus *Pseudomonas* C: Ordination of reads assigned to Pseudomonas at dilutions of 10^{-3} and above (the proposed limit of detection in this study, = 10^{2} cells per ml)
