# Supplementary material for: Novel application of metagenomics for the strain-level detection of bacterial contaminants within non-sterile industrial products – a retrospective, real-time analysis
Source: Microb Genom. 2022 Nov 24;8(11):mgen000884. doi: 10.1099/mgen.0.000884 (PMC9836090; doi:10.1099/mgen.0.000884)
Supplement: Supplementary material 2 [file mgen-8-884-s002.pdf]

Supplementary Table 1: Tabulated pairwise comparisons of popANI and shared genome coverage, corresponding to Figure 3 in the main manuscript. Key: Coverage overlap = The percentage of bases that are either covered or not covered in both of the profiles (Formula = length(coveredInBoth) / length(coveredInEither)); Compared base count = The number of considered bases; popANI = The Average Nucleotide Identity among compared bases between the two scaffolds (Formula = (compared_bases_count - population_SNPs) / compared_bases_count); Compared = percentage of the genome compared

**Sample 1**	**Sample 2**	**Coverage overlap (%)**	**Compared base count (bp)**	**popANI (%)**	**Compared (%)**
Cultivation-independent, 10^4 CFU/ml, Week 1	Cultivation-independent, 10^4 CFU/ml, Week 3	98.34	4777027	99.999	97.20
Cultivation-independent, 10^4 CFU/ml, Week 1	Cultivation-independent, 10^3 CFU/ml, Week 1	96.07	4665207	99.999	94.93
Cultivation-independent, 10^4 CFU/ml, Week 1	Cultivation-independent, 10^3 CFU/ml, Week 3	96.26	4667654	99.999	94.98
Cultivation-independent, 10^4 CFU/ml, Week 1	Cultivation-independent, 10^5 CFU/ml, Week 1	99.70	4861226	99.999	98.92
Cultivation-independent, 10^4 CFU/ml, Week 1	Cultivation-independent, 10^5 CFU/ml, Week 3	99.70	4860813	99.999	98.91
Cultivation-independent, 10^4 CFU/ml, Week 3	Cultivation-independent, 10^3 CFU/ml, Week 1	96.27	4629339	100.000	94.20
Cultivation-independent, 10^4 CFU/ml, Week 3	Cultivation-independent, 10^3 CFU/ml, Week 3	96.67	4638147	99.999	94.38
Cultivation-independent, 10^4 CFU/ml, Week 3	Cultivation-independent, 10^5 CFU/ml, Week 1	98.26	4777447	100.000	97.21
Cultivation-independent, 10^4 CFU/ml, Week 3	Cultivation-independent, 10^5 CFU/ml, Week 3	98.29	4777425	100.000	97.21
Cultivation-independent, 10^3 CFU/ml, Week 1	Cultivation-independent, 10^3 CFU/ml, Week 3	95.06	4544358	99.999	92.47
Cultivation-independent, 10^3 CFU/ml, Week 1	Cultivation-independent, 10^5 CFU/ml, Week 1	96.03	4666534	99.999	94.96
Cultivation-independent, 10^3 CFU/ml, Week 1	Cultivation-independent, 10^5 CFU/ml, Week 3	96.04	4666188	99.999	94.95
Cultivation-independent, 10^3 CFU/ml, Week 3	Cultivation-independent, 10^5 CFU/ml, Week 1	96.18	4667912	100.000	94.98
Cultivation-independent, 10^3 CFU/ml, Week 3	Cultivation-independent, 10^5 CFU/ml, Week 3	96.21	4667887	99.999	94.98
Cultivation-independent, 10^5 CFU/ml, Week 1	Cultivation-independent, 10^5 CFU/ml, Week 3	99.80	4865654	99.999	99.01

Supplementary Table 2: Coverage and popANI statistics for cultivation-independent sampling at 10^2 CFU/ml, during week 1. Key: Depth = depth of coverage (expressed as fold value); Breadth = percentage breadth of coverage; Median = median coverage; STD = standard deviation; SEM = Standard Error of the Mean; popANI = The Average Nucleotide Identity among compared bases between the two scaffolds (Formula = (compared_bases_count - population SNPs) / compared_bases_count)

**Depth (fold)**	**Breadth (%)**	**Median**	**STD**	**SEM**	**popANI (%)**
2.69	79.20	2	2.53	0.00115	99.999
